# Biomaterial-Driven Immunomodulation: Cell Biology-Based Strategies to Mitigate Severe Inflammation and Sepsis

**DOI:** 10.3389/fimmu.2020.01726

**Published:** 2020-08-04

**Authors:** Jackline Joy Martín Lasola, Henry Kamdem, Michael W. McDaniel, Ryan M. Pearson

**Affiliations:** ^1^Department of Microbiology and Immunology, University of Maryland School of Medicine, Baltimore, MD, United States; ^2^Department of Pharmaceutical Sciences, University of Maryland School of Pharmacy, Baltimore, MD, United States; ^3^Marlene and Stewart Greenebaum Comprehensive Cancer Center, University of Maryland School of Medicine, Baltimore, MD, United States

**Keywords:** nanoparticles, microparticles, biomaterials, innate immunity, macrophage, neutrophil, sepsis, inflammation

## Abstract

Inflammation is an essential component of a wide variety of disease processes and oftentimes can increase the deleterious effects of a disease. Finding ways to modulate this essential immune process is the basis for many therapeutics under development and is a burgeoning area of research for both basic and translational immunology. In addition to developing therapeutics for cellular and molecular targets, the use of biomaterials to modify innate and adaptive immune responses is an area that has recently sparked significant interest. In particular, immunomodulatory activity can be engineered into biomaterials to elicit heightened or dampened immune responses for use in vaccines, immune tolerance, or anti-inflammatory applications. Importantly, the inherent physicochemical properties of the biomaterials play a significant role in determining the observed effects. Properties including composition, molecular weight, size, surface charge, and others affect interactions with immune cells (i.e., nano-bio interactions) and allow for differential biological responses such as activation or inhibition of inflammatory signaling pathways, surface molecule expression, and antigen presentation to be encoded. Numerous opportunities to open new avenues of research to understand the ways in which immune cells interact with and integrate information from their environment may provide critical solutions needed to treat a variety of disorders and diseases where immune dysregulation is a key inciting event. However, to elicit predictable immune responses there is a great need for a thorough understanding of how the biomaterial properties can be tuned to harness a designed immunological outcome. This review aims to systematically describe the biological effects of nanoparticle properties—separate from additional small molecule or biologic delivery—on modulating innate immune cell responses in the context of severe inflammation and sepsis. We propose that nanoparticles represent a potential polypharmacological strategy to simultaneously modify multiple aspects of dysregulated immune responses where single target therapies have fallen short for these applications. This review intends to serve as a resource for immunology labs and other associated fields that would like to apply the growing field of rationally designed biomaterials into their work.

## Introduction

Inflammation is a complex and essential homeostatic response to extrinsic and intrinsic damage. This process is responsible for everything from recognition of microbial breeches into sterile sites and tissue damage to clearance of the insulting microbe and resolution of the immune response. Host defense mechanisms act to mobilize immune cells and molecules into vascularized tissues with the objective to eliminate the source of cell injury. Acute inflammation has been noted since antiquity by the cardinal signs of *rubor* (redness), *tumor* (swelling), *calor* (heat), *dolor* (pain), and *functio laesa* (loss of function) (1). These cardinal signs may also be accompanied by systemic features triggered by cytokine release, such as fever, changes in the peripheral white blood cell count, and increases in clinically detectable acute phase reactants in the blood stream (2). Given the vast coordination of tissue sites and organ systems, inflammation requires a finely tuned, highly regulated physiologic process where a concerted regulatory network of cellular and chemical mediators exists to limit the extent, severity, and spread of inflammation. Failure to effectively and efficiently resolve this process leads to a state of chronic inflammation that can exacerbate disease and pathologic processes. As the role of inflammation becomes further appreciated as a major driver of pathogenesis in many diseases, the need for technologies capable of modulating vascular and immune responses during uncontrolled inflammation will become increasingly necessary.

In this review, we will facilitate our discussion of dysregulated inflammatory responses within the context of severe inflammation and sepsis. These serve as fitting models for understanding the inflammatory response and what occurs when it fails to resolve as expected (3, 4). Unfortunately, there are currently very few nanotechnology platforms that specifically investigate their utility for this indication. As such, we will address what is known about the physiologic pathways and mechanisms at play during inflammation through discussions of emerging technological developments to modulate inflammation in a variety of diseases. This will serve as a starting point to consider future nanotechnology prospects to improve patient health for those suffering from sepsis.

Here, we will specifically focus on technologies where the immune responses can be attributed to the inherent physicochemical properties of the engineered biomaterial itself (i.e., in the absence of any immunomodulatory small molecule or biologic). Although the immune response has previously been treated as something that must be overcome in the development of nanoparticles and microparticles (henceforth, referred to collectively as nanoparticles) for clinical usage, the rise of immunotherapy for vaccine and immune tolerance applications has shed new light on the ways nanoparticle physicochemical properties can be used for similar purposes to modify existing immune responses (5–8), especially for indications where dysfunctional or exaggerated inflammation and immunological processes are contributing factors. Lastly, these nanotechnology-based approaches will be discussed in the context of what is known about the biological processes during inflammation as appreciation of nano-bio interactions (9, 10) allows for development of design strategies for future biomaterial approaches. Our objective is to highlight emerging patterns in biomaterial designs for a variety of nanoparticle platforms and how they have been shown to regulate multifactorial immune responses by acting at various points in the inflammatory cascade. Given that inflammation is exceptionally complex, we propose nanoparticles as a polypharmacological approach to shift how we think about therapeutics for severe inflammation and sepsis.

## The Persistence of Systemic Inflammation and Limitations of Sepsis Therapeutic Development

Inflammation is a powerful, multifactorial host defense mechanism intended to protect the host from microbial insult and tissue damage (11). As such, it is not only essential to the maintenance of homeostasis and protection but also can be deleterious on its own when regulatory mechanisms go awry. Examples of conditions characterized by dysregulated inflammatory responses include chronic inflammation, inappropriate fibrosis and scarring, and sepsis syndrome. These conditions demonstrate the delicate balance between damage and repair by which inflammation drives much of the pathology of common diseases including atherosclerosis, diabetes, neurodegenerative disorders, and others. Although situations of chronic inflammation and inappropriate wound healing are outside the scope of this review, sepsis syndrome provides a highly informative case study of what happens when acute inflammation continues unabated. When left unchecked, continuous inflammation leads to its own set of deleterious, systemic acute phase responses, and ineffectual regulatory responses.

Sepsis is a life-threatening condition of complex pathophysiological origin that develops due to an uncontrolled immune response during infection (12–14). Hallmark features of sepsis include profound acute pro-inflammatory cytokine responses, vascular endothelial leakage, and multi-organ failure (15). Concomitantly, a compensatory anti-inflammatory response develops in an attempt to resolve inflammation and promote tissue repair. This is accompanied by immune paralysis whereby antigen presenting cells (APCs) such as macrophages and dendritic cells lose their responsiveness to subsequent inflammatory challenge and significant alterations in immune cell apoptotic programming results in immunosuppression occurring with immune-mediated organ dysfunction (16–19). Beyond the dramatic physiologic changes, the human toll of sepsis and septic shock remains quite dramatic and accounted for over 2.5 million cases and $52 billion in aggregate costs between January 2010 and September 2016 alone (20). The global burden of sepsis is even greater with conservative estimates indicating that it is the leading cause of mortality and critical illness worldwide (13, 21). In 2017, it was estimated that 19.7% of all global deaths could be attributed to sepsis or sepsis-related causes (12). Because of this acute burden and the long-term physical, psychological, and cognitive disabilities for those who survive sepsis (22) efforts to improve treatment strategies and therapeutic approaches for those with and at risk of sepsis have been ongoing. While mainstays of treatment remain early administration of broad-spectrum antibiotics and intravenous fluids along with resuscitation, additional successful attempts to improve patient management for those with sepsis remain limited (23, 24).

Since the 1980's, over 100 therapeutic clinical trials have been conducted for sepsis and septic shock with little to no improved prospects for those affected. The general strategy for research and drug development within this space has been the generation of highly targeted agents that can be classified into categories based on known mechanisms at play in inflammation. These include as disparate and broad categories as anti-cytokines, anti-virulence factors, coagulopathy agents, anti-inflammatory agents, and even immune stimulators [reviewed nicely in (23–26)] with some of these examples being described in what follows. Of all the clinical trials, only activated protein C (drotrecogin alfa-activated), whose mechanism of action is an antithrombotic effect due to inhibition of factors Va and VIIIa in the coagulation cascade, was successfully licensed following a highly publicized clinical trial (27, 28). Yet, it was removed from the market a decade later due to further work showing not only a lack of a survival benefit for sepsis patients but also increased bleeding risks (29–32). With the inconclusive clinical trial data and controversial marketing strategies for drotrecogin alfa-activated, the example of activated protein C therapies and other failed clinical trials for targeted therapeutics raises the question, what are we missing in our strategies to treat sepsis? Various researchers have attempted to answer this question and have identified a few major themes (24–26): (1) preclinical models poorly recapitulate the complex physiological and molecular changes of sepsis syndrome; (2) patients with sepsis are plagued by a variety of initiating microbial infections and modes of entry; and, (3) patients are themselves very demographically complex based on age, sex, comorbidities, genetics, and infection site. However, one factor that remains underappreciated is the complex and redundant mechanisms at play to initiate the underlying bout of severe inflammation and the resultant sepsis. As such, any attempts to resolve the underlying dysregulated inflammation that triggers sepsis requires an approach that can address the redundancies of this highly coordinated defense mechanism. Elucidation of this multifactorial process requires ongoing work in preclinical models despite current recommendations to move away from such investigations (33).

Collectively, the efficacy of these single-agent, single-target therapeutics has not been as successful as preclinical models has suggested and when shown to be of some benefit, responses are highly dependent on their administration during a narrow treatment window with the associated immunosuppression rarely being addressed along with the vast pro-inflammatory response (24, 34). As such, safe and effective multi-targeted therapeutics for sepsis are critically needed to overcome the considerable heterogeneity of deficiencies at the cellular and molecular level that accumulate to result in deleterious tissue and multiorgan damage in sepsis. Modification of these vascular and immune cell responses using engineered nanoparticles is the basis for new therapies aimed to suppress inflammatory responses and functionally reprogram dysregulated cells and molecular pathways (7, 35, 36). In the following sections, we will describe approaches to treating severe inflammation and sepsis using nanoparticle strategies informed by the known cellular and molecular pathophysiology of inflammatory processes.

## Nanoparticle Modulators of Integrated Vascular and Immune Inflammatory Responses

**Figure 1:**
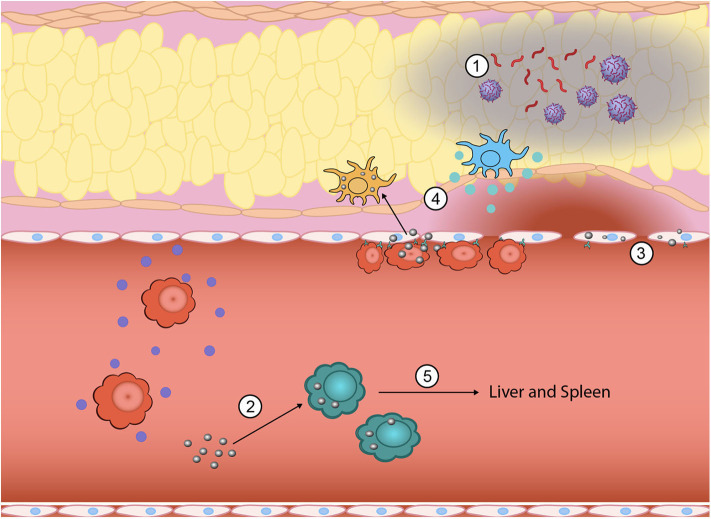
Inflammation is a highly complex, multistep process where nanoparticles can be engineered to intervene to tune the response at multiple points. During the initial generation of PAMPs and DAMPs, biomimetic nanoparticles have been used to sequester PAMPs and DAMPs from immune cell recognition (1). Innate immune cells that have taken up nanoparticles can be functionally reprogrammed from a pro-inflammatory phenotype (i.e., TNF-α, IL-1β, and IL-6 secreting) to an anti-inflammatory phenotype (2). The vascular endothelium also plays a key role in promoting inflammation and nanoparticles can be used to downregulate attachment of circulating immune cells and subsequent exudation (3). Nano-bio interactions can also alter direct homing to inflamed tissue sites by either eliminating chemokine production at the site (4) or redirecting inflammatory cells away from the inflamed site to the liver and spleen for elimination (5).

As a highly regulated process, deficient and/or overexaggerated responses at any step in the inflammatory cascade can result in serious deterioration in the health status of an individual. The inflammatory response can be characterized by the following processes: (1) recognition of the injurious agent, (2) regulation of the response (control), (3) recruitment of leukocytes, (4) removal of the agent, and (5) resolution (repair) (11). Throughout this process, microvascular tissue, innate immune cells, and circulating soluble mediators act to respond. Further, deficits in the adaptive immune system can contribute to the body's inability to control the infection and repair. Within each step of this response, points for intervention exist for therapeutics to alter the progression of inflammation and modulate the systemic responses (Figure 1). For decades, researchers have focused on developing single molecule or single pathway targeted therapeutics to modify highly specific regulatory nodes of the inflammatory response. As our discussion progresses, a number of these approaches will be discussed to compare and contrast with newer nanotechnologies driven by current biological understanding of inflammation. By evaluating the numerous approaches, the intention is to suggest future paths of therapeutic research and development to alter outcomes for those with severe inflammation and sepsis.

### Halting Inflammation Before It Starts: Strategies to Sequester the Initiating Warning Signals of Inflammation

Initiation of inflammation requires recognition of the infectious microbe or products of cell and tissue damage. This work is accomplished by tissue macrophages, dendritic cells, and mast cells of the innate immune system, in addition to other sentinel cells resident in tissues that contain pattern recognition receptors (PRRs). These PRRs are unique in that they can recognize pathogen-associated and damage-associated molecular patterns (PAMPs and DAMPs, respectively) in a manner that triggers general molecular warning programs to initiate protective processes against the inciting insults (37, 38). These receptors include Toll-like receptors (TLRs) on the plasma and endosomal membranes, C-type lectin receptors (CLRs) on the plasma membrane, NOD-like receptors (NLRs) on the plasma and endosomal membranes and in the cytoplasm, RIG-I-like receptors (RLRs) in the cytoplasm, and AIM2-like receptors (ALRs) in the cytoplasm and nucleus (39). These receptors are unique in that they recognize cellular products exclusively produced by microbes, such as lipopolysaccharide (LPS) from Gram-negative bacteria or double-stranded RNA from specific viral classes, or cellular components that are only released by the host during times of tissue and cellular damage like high mobility group box 1 (HMGB1) (40, 41). These cellular responses are complemented with circulating proteins that serve as complementary humoral responses. Antibodies, complement proteins (42), and collectins (43–45) also recognize microbes, opsonize them, and target them for ingestion by phagocytes and activation of other immune cells via Fc receptors.

Given that inflammation starts with recognition of these PAMPs, DAMPs, and microbes, at the nascent site of inflammation, limiting the initiation of this process serves as a potential strategy by which to limit the severity of inflammation and halt progression to systemic inflammation. Traditional strategies to halt these initial stages of the inflammatory cascade have focused on neutralizing microbes, such as continuing antibiotic development or even utilization of human antiserum against microbes, such as against *Escherichia coli* (46). Newer approaches aim to bind and neutralize PAMPs, such as the development of monoclonal antibodies targeting the lipid A moiety of LPS (47–50) or direct antagonizing of the PRRs like trials conducted with eritoran, a TLR4 antagonist derived from lipid A (51, 52), or small molecule inhibitors of TLR signaling like TAK-242 (53).

**Figure 2:**
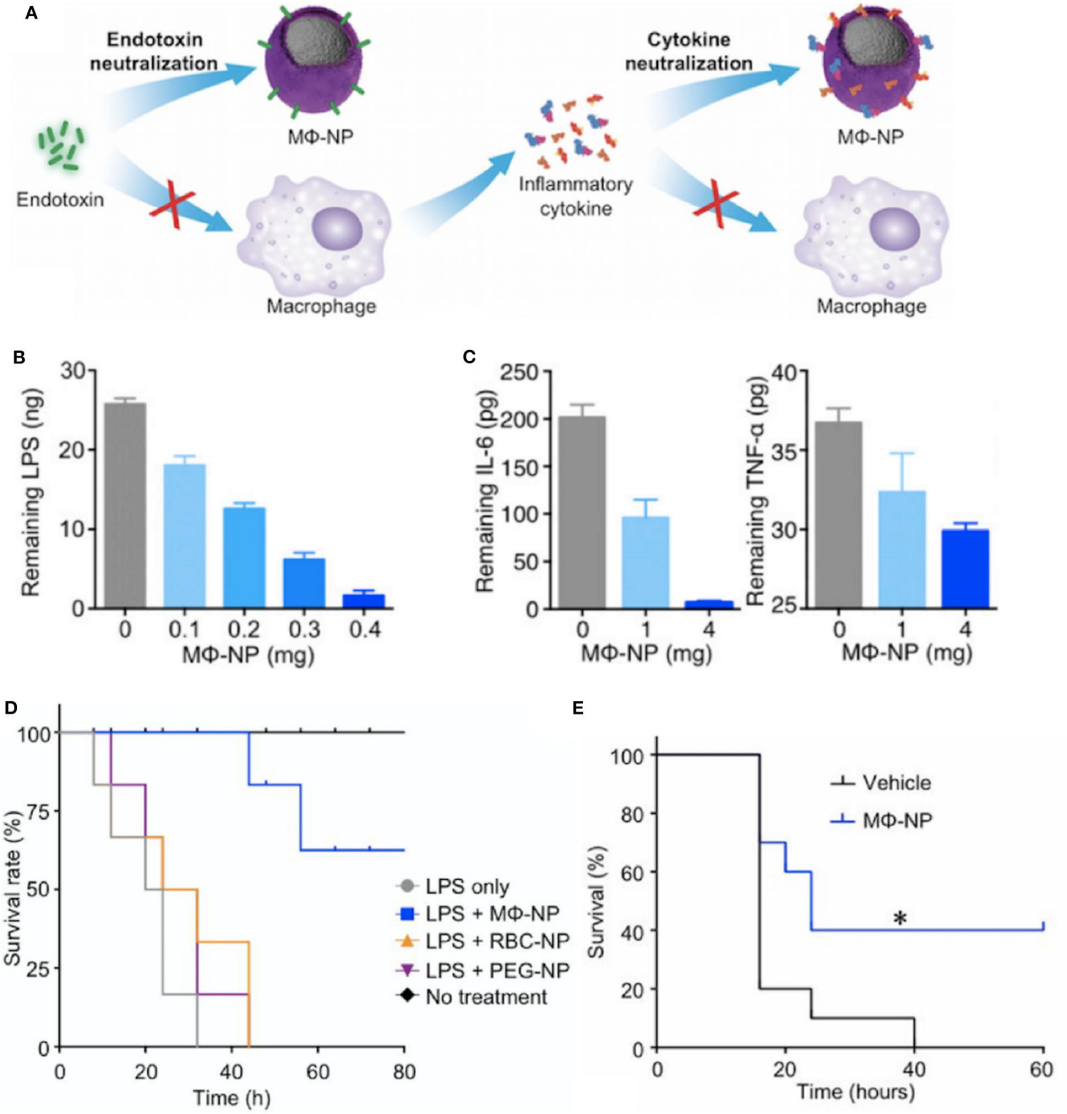
Macrophage mimicking nanoparticles (MΦ-NP) sequester bacteria derived endotoxin and subsequent inflammatory cytokines to limit inflammation associated damage **(A)**. This results in a dose-dependent ability of the MΦ-NP to reduce free LPS **(B)** and pro-inflammatory cytokines such as IL-6 and TNF-α **(C)** *in vitro*. LPS-induced endotoxemia **(D)** and *E. coli* bacteremia **(E)** show a survival benefit specific to the biomimetic MΦ-NP, where *P < 0.05. Adapted from (54). Copyright (2017) National Academy of Science.

More recent biomaterial strategies to prevent this initial recognition of microbial products and their ensuing damage are notable for biomimetic approaches (Figure 2 and Table 1) to sequester these initiators of inflammation and halt the cascade before it begins. Kunz et al. (55) developed cell-derived nanoparticles (CDNPs) to limit inflammation and showed that the CDNP platform was able to limit bacterial growth *in vitro*. CDNPs were generated via high-speed centrifugation of fibroblast cytoplasmic contents to isolate the desired exosomes. These exosomes were largely composed of proteins such as annexin A5, heat shock proteins, peroxiredoxines, with small traces of DNA and RNA, that showed preferential uptake by neutrophils, inflammatory monocytes, and macrophages, all key cells for the initiation of inflammation. This was correlated with decreased IL-6 levels in the peritoneum of mice with cecal ligation and puncture (CLP)-induced polymicrobial sepsis. Additional work showed that in an *in vitro* system, coincubation of these CDNPs with *Pseudomonas aeruginosa* resulted in direct decreases in bacterial colony-forming units, suggesting an additional bactericidal effect of the CDNPs. To contrast, a separate strategy by Thamphiwatana et al. (54) used a similar strategy of isolating immune cell components to drive protective responses against inflammation. The authors used poly(lactic-*co*-glycolic acid) (PLGA) as the core polymer for the particles and coated these particles with macrophage-derived cell membranes to prepare macrophage mimicking nanoparticles. As described in Figure 2, the authors show using both macrophages and endothelial cells (HUVECs), the ability of these macrophage mimicking nanoparticles to sequester LPS away from the PRRs of cells necessary to initiate the inflammatory cascade with an additional effect of also sequestering away inflammatory cytokines to prevent further inflammatory activation of macrophages and HUVECs. Using LPS-induced endotoxemia and *E. coli*-induced bacteremia murine models, these particles were shown to have a survival benefit specifically linked to the inclusion of the macrophage membranes in the particle formulation.

**Table 1:** Strategies to sequester the initiating warning signals of inflammation.

**Biological effect**	**Physicochemical properties**				**Biological models**		**References**
	**Materials**	**Size**	**Zeta potential/charge**	**Surface coating**	**Cell type**	**Animal models**	
**BIOMIMETIC STRATEGIES TO HALT INITIATION OF INFLAMMATION**							
Decreased bacterial growth leading to decreased PAMP/DAMP availability	MC3T3-E1 fibroblast-derived annexins (abundant annexin A5), actin, histones, heath shock proteins, myosin, peroxiredoxines, vimentin; small traces of nucleic acids	50–200 nm			Neutrophils, inflammatory monocytes, macrophages	Mice	(55)
Sequestration of LPS and inflammatory cytokines	Poly(lactic-*co*-glycolic acid)	100–105 nm	−30 to −23 mV	Macrophage cell membrane	J774 macrophages, human umbilical vein endothelial cells	Mice (C57Bl/6, BALB/c)	(54)
**SEQUESTRATION OF COMPLEMENT PROTEINS**							
Sequestration of circulating complement proteins triggering opsonization	Poly(lactic-*co*-glycolic acid)	40–50 nm	−70 to −50 mV	Poly(ethylene glycol) (PEG_550_ and PEG_2000_)	J774 macrophages		(56)
Sequestration of circulating complement proteins triggering opsonization	Multi-walled carbon nanotubes	0.4–4.2 nm (length), 12–34 nm (diameter)		Carboxylmethyl cellulose	U937 monocytes, human peripheral blood monocytes		(56)
Sequestration of complement proteins triggering opsonization	Multi-walled carbon nanotubes	0.4–4.2 nm (length), 12–34 nm (diameter)		RNA	U937 monocytes, human peripheral blood monocytes		(56)

As noted above, in addition to direct cellular recognition of PAMPs and DAMPs, circulating proteins of the innate immune response can trigger activation of inflammatory pathways. Of particular note are those within the complement pathway where C3a, C4a, and C5a, serve as triggers of anaphylaxis and chemotaxis. Because of their key role as humoral mediators of inflammation, the interaction between biomaterials and the complement pathway are of great interest. In one study, PLGA particles were shown to trigger differential levels of complement activation based on the molecular weights of the poly(ethylene glycol) surface coating. By combining PEG_550_ with PEG_2000_ as the surface coating of PLGA particles, Pannuzzo et al. were able to limit generation of C5a and downstream complement components without altering particle uptake by macrophages (56). Another platform showed that multi-walled carbon nanotubes (CNTs) surface modified with carboxymethyl cellulose (CMC-CNT) or RNA (RNA-CNT) appear to serve as a type of sink for deposition of complement pathway proteins. This has the net effect of modifying the inherent pro-inflammatory responses of CNTs through analysis of dampened transcription of TNF-α and IL-1β in macrophages (57). In contrast, a study of CNTs showed that in combination with LPS activation, the pro-inflammatory effects of CNTs were mediated through inflammasome activation (58). This emphasizes the tunability of the immune response to CNTs in a manner dependent on their physicochemical properties. These varying responses to CNTs, with a particular emphasis on their effects on complement are described in a recent review (59).

### Regulating the Regulators: Altering Production of Molecular Mediators of Inflammation

Due to its destructive potential, tight regulation of the initiation and progression of inflammation by its mediators is essential to limit deleterious effects beyond those necessary for eliminating the initial offending agent. As such, these mediators—including histamine, prostaglandins and leukotrienes, and cytokines and chemokines—are often targeted therapeutically to limit inflammation during disease processes (60, 61).

The vasoactive amine histamine is stored preformed in cells and is released upon mast cell degranulation (also blood basophils and platelets). This release allows for binding to the H_1_ receptor of microvascular endothelial cells to trigger arteriole dilation and increased venule vascular permeability. Due to histamine activity, it commonly serves as an anti-inflammatory target, particularly for allergy, and H_1_ receptor antagonists like diphenhydramine, loratadine, and cetirizine, are some of the most commonly used drugs for managing allergic reactions and acute inflammatory processes (62).

Arachidonic acid (AA) is found in membrane phospholipids and can be released from the membrane phospholipids (particularly from phospholipase A_2_, PLA_2_) upon activation to produce interesting classes of inflammation mediators, prostaglandins (PGs) and leukotrienes. Cyclooxygenases (COX-1 and COX-2) in mast cells, macrophages, and endothelial cells produce PGs to trigger vascular and systemic signs and symptoms of inflammation (63). PGE_2_ and PGD_2_ (mast cells) trigger vasodilation and increases permeability of postcapillary venules to allow for edema formation, whereas PGF_2a_ stimulates uterine, bronchial, and small arteriole smooth muscle contraction. Prostacyclin (PGI_2_) is produced in vascular endothelium and serves as a vasodilator and potent inhibitor of platelet aggregator, in addition to serving as a potentiator of other mediators that increase vascular permeability and chemotaxis to sites of injury. Thromboxane (TxA_2_), produced in platelets, opposes the effects of prostacyclin in that it is a vasoconstrictor and a potent inducer of platelet aggregation. In addition to these local effects, prostaglandins are implicated in promoting the systemic symptoms of inflammation, namely pain and fever. In contrast, leukotrienes are produced in leukocytes and mast cells by lipoxygenases where LTB_4_ serves as a potent chemoattractant while LTC_4_, LTD_4_, and LTE_4_, serve to induce vasoconstriction, bronchospasm, and increased permeability of venules in a manner more potent than the initial histamine release from mast cell degranulation (64).

Given the central role of AA metabolites in inflammation, pharmacologic inhibitors of AA metabolism are widespread in the pharmacopeia. Corticosteroids are an essential class of drugs that can prevent the initial release of AA by phospholipase activity in addition to a series of other proposed mechanisms of action. Non-steroidal anti-inflammatory drugs (NSAIDs) like naproxen and ibuprofen are common over the counter and prescription medication that serve as COX inhibitors to limit inflammation, while lipoxygenase inhibitors and leukotriene receptor antagonists serve as therapeutic strategies in asthma management due to their specific induction of bronchial smooth muscle contraction (63).

In contrast to the non-specific, broad activities of corticosteroids or the highly specific COX inhibitors utilized for asthma management, the applicability of these strategies for severe inflammation and sepsis have been of limited utility. A variety of clinical trials using corticosteroids have shown inconclusive results ranging from benefit with hydrocortisone and fludrocortisone (65) to no overall effect with hydrocortisone (66–70), methylprednisolone, or dexamethasone (71) with some clinical studies concluding corticosteroid strategies with methylprednisolone to actually be a detriment to survival (72). Similarly, a trial for ibuprofen, a common NSAID that serves as an unselective COX inhibitor, showed no effect on mortality in severe sepsis (73).

**Figure 3:**
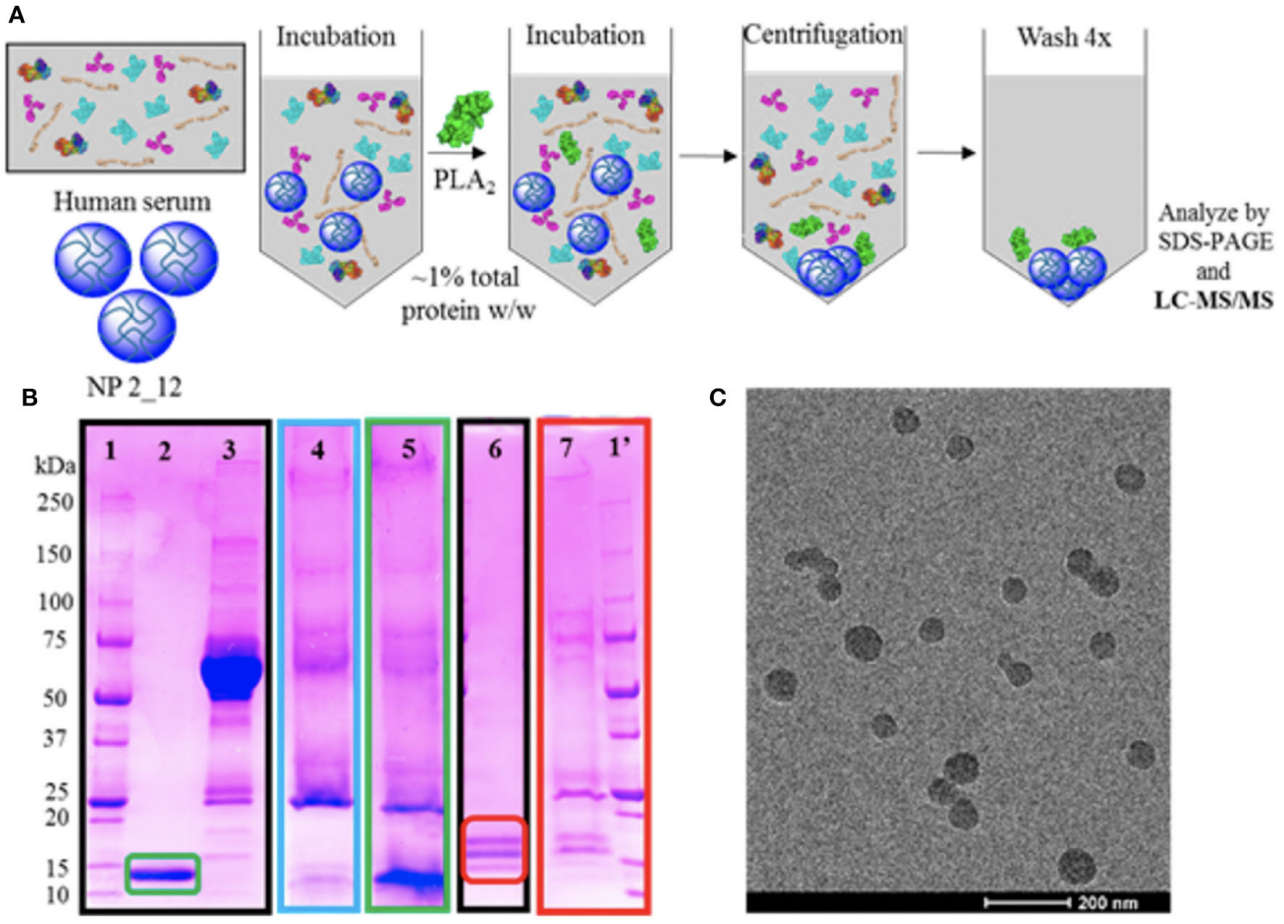
Selectivity experiments and TEM characterization of nanoparticles for targeted sequestration of venom proteins. Polymer composition was optimized to enable specificity toward venom yet avoid serum protein binding. Strategy for assessing selectivity of nanoparticles to venom **(A)**. Selectivity assessment via SDS-PAGE visualization **(B)** of (1/1′) ladder; (2) purified PLA_2_ from *Naja mossambica* venom; (3) serum control; (4) nanoparticle in serum only; (5) nanoparticle incubated in serum and PLA_2_ from *N. mossambica* venom; (6) purified PLA_2_ from honey-bee venom; (7) nanoparticle incubated in ovine plasma and PLA_2_ from honey-bee venom. Unstained TEM image of nanoparticle for sequestration of venom **(C)**. Reprinted with permission from (74). Copyright (2016) American Chemical Society.

The limited successes in utilizing these anti-inflammatory strategies in sepsis, has left open the opportunity for biomaterials to serve a role in modifying these immune mediators. Often, due to the inherent capability of materials to be easily altered to better bind a broad variety of metabolites in the bloodstream. An interesting approach is one taken by O'Brien et al. (74, 75) where poly(*N*-isopropylacrylamide) (NIPAm) particles generated in combination with other acrylamide moieties were synthesized to alter the affinity of the protein corona for a variety of plasma components (Figure 3). This desire to “tune” the corona for high affinity and selectivity to a variety of biomacromolecules showed that although NIPAm-based particles showed little affinity for plasma proteins, the hydrophobicity of NIPAm-based particles allowed for them to interact favorably with lipophilic molecules. This later was used to show that they could be used for lipid-bound toxin sequestration and neutralization, such as whole honey-bee venom containing a significant amount of venomous PLA_2_. It would be interesting to see this work expanded to see if this sequestration and neutralization strategy via protein corona tuning could be applied to neutralizing endogenous lipid species released during inflammation such as the AA metabolites described above that are produced upon vascular endothelial activation.

Similar to the role played by AA metabolites in regulating vascular activity, cytokines and chemokines are proteins produced and secreted by a variety of cell types (activated lymphocytes, macrophages, dendritic cells; also, endothelial, epithelial, and connective tissue cells) to regulate immune and inflammatory activity. In acute inflammation, tumor necrosis factor alpha (TNF-α) and interleukin-1 beta (IL-1β) are essential for leukocyte recruitment by their promotion of endothelial adhesion and diapedesis. Given this activity, research on cytokine receptor blockade has produced therapeutic drug classes that have broad effects at modifying autoimmune disease outcomes. Beyond activating the endothelium and leukocytes, TNF-α, IL-1β, and IL-6 combine to induce the systemic acute phase response that is implicated in the development of sepsis (76–78). In comparison, chemokines serve to attract specific cells to the inflammatory site with individual chemokines of note being IL-8 from macrophages and endothelial cells that drive neutrophil recruitment (79), while monocyte chemoattractant protein (MCP-1) (80), macrophage inflammatory protein-1α (MIP-1α) (81), and regulated and normal T cell expressed and secreted (RANTES) (82), attract monocytes, eosinophils, basophils, and lymphocytes.

The elucidation of molecular mechanisms involved in leukocyte recruitment and migration during inflammation have led to major developments in the generation of therapeutic targets for a variety of inflammation-mediated diseases. Although first discovered to play a role in the pathogenesis of sepsis (83, 84), therapies to directly block TNF-α and IL-1β signaling have done more to change the progression and day-to-day symptomatology for patients with a variety of autoimmune (60, 85) and dermatological conditions (86). In contrast, a variety of clinical trials utilizing anti-TNF monoclonal antibodies (26) or even fusion proteins to neutralize TNF-α, like lenercept (87, 88), or etanercept (89), which is in common clinical usage today, have shown no benefit and even harm for patients with septic shock in the case of etanercept (89). This has also been shown with anakinra, an IL-1β decoy receptor, which failed to conclusively show a survival benefit for patients with sepsis or septic shock (90–92).

**Figure 4:**
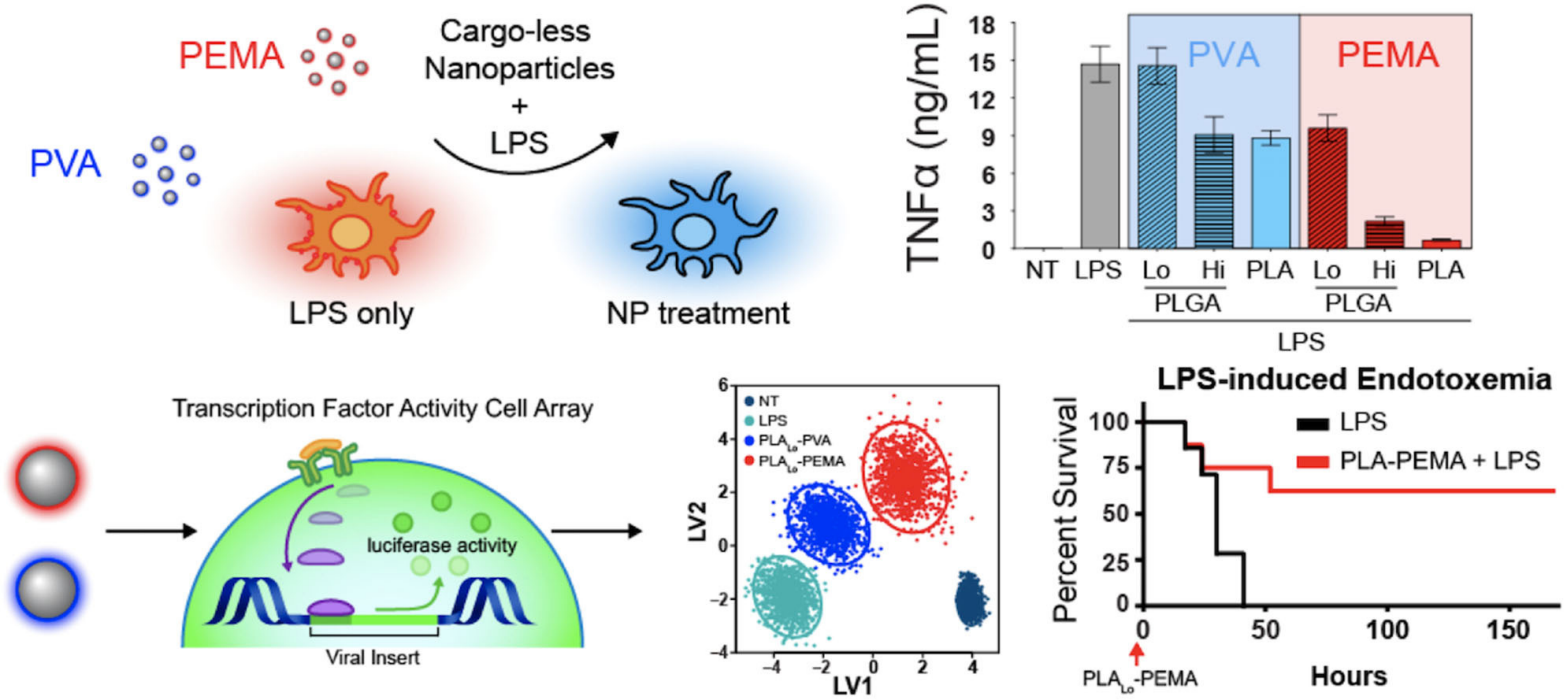
Immunomodulatory effects of nanoparticles. Nanoparticle-dependent inflammatory cytokine suppression of innate immune cells when stimulated with LPS (top). Dynamic transcription factor activity of bone marrow-derived macrophages treated with particles followed by LPS stimulation and improved survival in lethal LPS-induced endotoxemia model. PVA, neutral charge. PEMA, negative charge. Adapted from (93). Copyright (2019) Elsevier.

Interestingly, biomaterials have also been shown to have inherent capabilities to alter immune cells to downregulate key chemotactic molecules (Figure 1 and Table 2) at play in recruiting leukocytes to inflammatory sites. As described above, key players of this process include the cytokines TNF-α and IL-1β. Inhibition of the innate immune cell's capability to secrete TNF-α and IL-1β serves to achieve a similar end as halting initiation of inflammatory signaling and shows the ability to act following activation of inflamed immune cells. Multiple groups have shown a capability to utilize biomaterials to affect this alteration in a variety of inflammation models and suggest a diversity of strategies to impart a similar net effect to modify the molecular regulators of immune activity during inflammation. Using poly(lactic acid) (PLA) and PLGA as nanoparticle cores with poly(ethylene-*alt*-maleic acid) (PEMA) and poly(vinyl alcohol) (PVA) as surfactants, Casey et al. (93), showed that polymer-based biomaterials, lacking any small molecule or biologic for therapeutic effect, have the inherent capability of suppressing cytokine secretion from bone marrow-derived macrophages and dendritic cells following LPS or CpG-DNA stimulation (Figure 4). This effect occurred in a surface charge-dependent manner and used polymeric particles in the 350–500 nm diameter range. Furthermore, these materials imparted a survival benefit in a murine LPS-induced endotoxemia model for sepsis. Remarkably, similar results were observed using completely different material composition. In another study, 2 nm gold core nanoparticles with a surface coating of hydroxylated tetraethylene glycol (TEGOH) (98)—again without any delivery of small molecules, peptides, or nucleic acid products—showed a survival benefit in a sepsis model. These nanoparticles were characterized as having an overall net neutral charge and the *in vitro* suppression of TNF-α production in monocytes appeared regardless of choice of surfactant (the TEGOH described above or the hydrophobic tetraethylene glycol coating, ZDiPen). Interestingly, using a murine LPS-induced endotoxemia model showed that only the TEGOH-coated gold nanoparticles showed the similar suppression of TNF-α production, whereas the hydrophobic ZDiPen failed to recapitulate the cytokine response. This further demonstrates the importance of designing nanoparticles with appropriate physicochemical properties followed by relevant *in vitro* and *in vivo* testing to obtain a comprehensive understanding of their effects on desired immunological outcomes.

**Table 2:** Methods to alter cytokine availability.

**Biological effect**	**Physicochemical properties**					**Biological models**		**References**
	**Materials**	**Size**	**Zeta potential/charge**	**Surface coating**	**Functionalization**	**Cell type**	**Animal models**	
Decreased secretion of IL-6, TNF-α	Poly(lactic acid)	350–500 nm	−50 to −40 mV	Poly(ethylene-*alt*-maleic acid)		Bone marrow-derived macrophages	Mice (C57BL/6)	(93)
Partial decreased secretion of IL-6, TNF-α	Poly(lactic acid)	350–500 nm	−25 mV	Poly(vinyl alcohol)		Bone marrow-derived macrophages	Mice (C57BL/6)	(93)
Sequestration of IL-6, TNF-α, IFNɤ; decreased serum IL-6, TNF-α, IFNɤ	Poly(lactic-*co*-glycolic acid)	100–105 nm	−30 to −23 mV	Macrophage cell membrane		J774 macrophages, human umbilical vein endothelial cells	Mice (C57BL/6, BALB/c)	(54)
Decreased secretion of TNF-α, IL-6; increased serum IL-10; decreased serum IL-6	Poly(lactic-*co*-glycolic acid)	140–165 nm	0 to 0.5 mV		di(α2 → 8)*N*-acetylneuraminic acid	Peritoneal macrophages	Mice (C57BL/6)	(94)
Decreased secretion of IL-6, TNF-α	Poly(lactic-*co*-glycolic acid)	350–500 nm	−50 to −40 mV	Poly(ethylene-*alt*-maleic acid)		Bone marrow-derived macrophages	Mice (C57BL/6)	(93)
Partial decreased secretion of IL-6, TNF-α	Poly(lactic-*co*-glycolic acid)	350–500 nm	−25 mV	Poly(vinyl alcohol)		Bone marrow-derived macrophages	Mice (C57BL/6)	(93)
Increased CD206, IL-10, and arginase 1	Poly(lactic-*co*-glycolic acid)	350–500 nm	−50 to −40 mV	Poly(ethylene-*alt*-maleic acid)		Macrophages	Mice (C57BL/6)	(95)
Increased expression of *IL1RN, IL10*	Poly(phosphorHydrazone)				Acid azabisphosphorous	Monocytes	Human volunteers, cynomolgus monkeys	(96, 97)
Decreased TNF-α production *in vitro*; decreased serum TNF-α following LPS-induced endotoxemia	Gold	2 nm (core)	Neutral charge	Tetraethylene glycol with end hydroxyl group (TEGOH)		Monocytes	Mice	(98)
Decreased TNF-α production *in vitro*; increased serum TNF-α following LPS-induced endotoxemia	Gold	2 nm (core)	Neutral charge, hydrophobic	Tetraethylene glycol with hydrophobic end group (ZDiPen)		Monocytes	Mice	(98)
Decreased secretion of IL-1β, TNF-α, IL-6, IL-8	Gold	5 nm		Inner lipid: 1,2-dipalmitoyl-*sn*-glycero-3-phosphoethanolamine-N-[3-(2-pyridyldithio)propionate] (PDP PE 16:0) or 1,2-dioleoyl-*sn*-glycero-3-phosphoethanolamine-N-[3-(2-pyridyldithio)propionate (PDP PE 18:1) Outer lipid: 1,2-dipalmitoyl-*sn*-glycero-3-phsphocholine (DPPC), spingomyelin, cardiolipin, 1,2-dilinoleoyl-*sn*-glycero-3-phospho-(1′-rac-glycerol) (18:2 PG), 1,2-dimyristoyl-*sn*-glycer-3-phosphoethanolamine-N-(lissamine rhodamine B sulfonyl) (14:0 Liss Rhod PE)		Monocytes	Human	(99)
Decreased expression of *Il6, Il1b, Tnf*	Hydroxylated fullerene (C_60_[OH]_44_)					Peritoneal macrophages	Mice (C57BL/6)	(100)
Decreased TNF-α, IL-1β secretion and increased IL-10 secretion	Nanodiamond	5 nm	Negatively charged		Octadecylamine	Macrophages	Human	(101)
Decreased peritoneal IL-6 and IL-10 following CLP	Cell-derived nanoparticle (CDNPs)—composed of annexins, actin, histones, heat shock proteins, myosin, peroxiredoxines and vimentin and small traces of nucleic acids, with annexin A5 (AnxA5) being one of the most abundant components; [protein] = 150 μL/mL, [DNA] = 2 μg/mL, [RNA] = 4 μg/mL	50–200 nm				Source of CDNPs: MC3T3-E1 fibroblast cells, peritoneal lavage Takes up CDNPs: neutrophils, inflammatory monocytes, macrophages	Mice	(55)

### Recruiting Leukocytes to Inflammatory Sites: Biomaterial-Driven Modification of Cellular Trafficking Patterns

A major role of lipid and peptide mediators for inflammation is to facilitate the recruitment of leukocytes to the sites of inflammation, which is necessary to eliminate the source of the infection and/or tissue damage. However, infiltration of these leukocytes can also further injure the inflamed tissue or nearby healthy tissue through the inherent activity of the neutrophils, inflammatory monocytes, macrophages, and other leukocytes. In order to limit their damage at the inflamed site beyond what is needed to control the infection or initial tissue damage, understanding how these sites are accessed from the vasculature and modification of the accessibility of these tissues can be of benefit in developing therapeutic strategies (Figure 1 and Table 3).

**Table 3:** Modifiers of cellular trafficking patterns.

**Biological effect**	**Physicochemical properties**				**Biological models**		**References**
	**Materials**	**Size**	**Zeta potential/charge**	**Surface coating**	**Cell type**	**Animal models**	
**DIRECT MODIFICATIONS OF INTEGRIN AND SELECTIN AVAILABILITY**							
Reduced neutrophil adhesion and migration across the endothelium to limit vascular inflammation modulated via adherent neutrophils; protective against lung injury with Syk inhibitor to prevent inside-out signaling	Polystyrene	100–117 nm		Denatured albumin	Neutrophils	Mice	(102, 103)
Binds ICAM-1 on HUVEC to deliver NF-κB inhibitors and stop diapedesis	Cell membrane-formed nanovesicles (cell disruption by nitrogen cavitation, centrifugation, and extrusion—contains integrin β2	200 nm	−20 to −12 mV		Source of nanovesicles: HL 60 myeloid cells		(104)
**DECREASED CHEMOATTRACTANTS TO INFLAMED SITE**							
Decreased secretion of MCP-1	Poly(lactic acid)	350–500 nm	−50 to −40 mV	Poly(ethylene-*alt*-maleic acid)	Bone marrow-derived macrophages	Mice (C57BL/6)	(93)
Partial decreased secretion of MCP-1	Poly[lactic acid)	350–500 nm	−25 mV	Poly(vinyl alcohol)	Bone marrow-derived macrophages	Mice (C57BL/6)	(93)
Decreased secretion of MCP-1	Poly(lactic-*co*-glycolic acid)	350–500 nm	−50 to −40 mV	Poly(ethylene-*alt*-maleic acid)	Bone marrow-derived macrophages	Mice (C57BL/6)	(93)
Partial decreased secretion of MCP-1	Poly(lactic-*co*-glycolic acid)	350–500 nm	−25 mV	Poly(vinyl alcohol)	Bone marrow-derived macrophages	Mice (C57BL/6)	(93)
Decreased secretion of IL-8, CCL5/RANTES, and CCL2/MCP-1	Gold	5 nm		Inner lipid: 1,2-dipalmitoyl-*sn*-glycero-3-phosphoethanolamine-N-[3-(2-pyridyldithio)propionate] (PDP PE 16:0) or 1,2-dioleoyl-*sn*-glycero-3-phosphoethanolamine-N-[3-(2-pyridyldithio)propionate (PDP PE 18:1) Outer lipid: 1,2-dipalmitoyl-*sn*-glycero-3-phsphocholine (DPPC), spingomyelin, cardiolipin, 1,2-dilinoleoyl-*sn*-glycero-3-phospho-(1′-rac-glycerol) (18:2 PG), 1,2-dimyristoyl-*sn*-glycer-3-phosphoethanolamine-N-(lissamine rhodamine B sulfonyl) (14:0 Liss Rhod PE)	Monocytes	Human	(99)
Decreased CCL2 and CCL4 production	Gold	13–20 nm	Negative charge	Peptides with hydrophobic and aromatic residues	Monocytes		(105, 106)
**DIVERSION OF INFLAMMATORY CELLS AWAY FROM SITE OF INFLAMMATION**							
Negligible sequestration of inflammatory monocytes and neutrophils away from sites of inflammation	Poly(lactic acid)	430–470 nm	−47 to −31 mV	Poly(ethylene-*alt*-maleic acid)	Inflammatory monocytes, neutrophils	Mice (SJL/J)	(107)
Negligible sequestration of inflammatory monocytes and neutrophils away from sites of inflammation	Poly(lactic-*co*-glycolic acid)—low molecular weight	330–510 nm	−56 to −40 mV	Poly(ethylene-*alt*-maleic acid)	Inflammatory monocytes, neutrophils	Mice (SJL/J)	(107)
Sequestration of inflammatory monocytes and neutrophils away from sites of inflammation	Poly(lactic-*co*-glycolic acid)—high molecular weight	510–590 nm	−43 to −36 mV	Poly(ethylene-*alt*-maleic acid)	Inflammatory monocytes, neutrophils	Mice (C57BL/6, SJL/J, BALB/c)	(107, 108)
Sequestration of inflammatory monocytes away from sites of inflammation	Polystyrene	500 nm	Negatively charged		Inflammatory monocytes	Mice (C57BL/6, SJL/J, BALB/c)	(108)

Initiation of removal of the injury source requires dilation of the small blood vessels to allow for increased blood flow, increased permeability of the microvasculature, and emigration of the leukocytes from the microcirculation to accumulate in the inflamed tissue (109). Recruitment of leukocytes involves interactions between the vasculature and the immune response. Interjection at any of these steps through altered signaling and inhibition of chemical mediators or redirection and sequestration of leukocytes can alter the course of inflammation to limit the damage it inflicts.

Following sentinel cell recognition of breaches of normal tissue, the vascular mediators described above are mobilized to induce vasodilation while other chemical mediators trigger increased permeability of the postcapillary venules (110). This increased vessel diameter and loss of fluid slows blood flow and allows for concentration of blood cells at the site of tissue injury. As the stasis matures, it is accompanied by increasing amounts of immune mediators at the site to aid in exudation of leukocytes. Additionally, the vascular endothelium expresses increased levels of adhesion molecules that allow for leukocytes, particularly neutrophils, to accumulate along the endothelium and allow for emigration from the vasculature into the injured tissue.

Activation of the vascular endothelium results in selectin upregulation mediated by release of cytokines and chemokines by tissue macrophages, mast cells, and endothelial cells in response to injury. In particular, TNF-α and IL-1β act on endothelial cells of the postcapillary venules near the infection to trigger expression of E-selectin and ligands for L-selectin. Histamine and thrombin also play a role and stimulate P-selectin redistribution from endothelial cell granules (Weibel-Palade bodies) to the cell surface. In contrast, firm adhesion is mediated by integrins whose expression are also under the influence of TNF-α and IL-1β. In the case of endothelium, these cytokines induce expression of vascular cell adhesion molecule 1 (VCAM-1, the ligand for β1 integrin VLA-4) and intercellular adhesion molecule 1 (ICAM-1, the ligand for the β2 integrins LFA-1 and Mac-1). Under normal conditions, the binding of the integrins to VCAM-1 and ICAM-1 are relatively low affinity, but under the influence of cytokines binding to the rolling leukocytes, VLA-4 and LFA-1 are converted to a high affinity state that allows for firm binding of the leukocytes to the endothelial surface (111).

Within the field of biomaterials, numerous groups have attempted to alter these vascular interactions to reduce inflammation [reviewed nicely in (112, 113)] but most have focused on delivery of therapeutics rather than investigation of physicochemical interactions that may modify endothelial behavior. In a few notable examples (102, 103), polystyrene in combination with denatured albumin modulated neutrophil adherence to the vasculature. This interaction aided in delivery of Syk inhibitors to prevent the inside-out signaling that increases leukocyte adhesion to the endothelium. By altering cellular trafficking through making the endothelium less sticky, these studies showed a protective effect against lung injury mediated through alterations in neutrophil activity. Similarly, direct delivery of NF-κB inhibitors to could be achieved through a biomimetic approach. Gao et al. (104) used myeloid cell-derived nanovesicles containing β2 integrins to bind directly to ICAM-1 on HUVECs. This showed a two-fold effect by firstly physically blocking further binding of other leukocytes while also delivering NF-κB inhibitors at the site to stop additional leukocyte diapedesis across the endothelium. Thus, developing a method to use inflammatory cell derivatives to block recruitment of leukocytes is a strategy similar in concept to that of cell membrane-coated nanoparticles described above by Thamphiwatana et al. (54).

Prevention of leukocyte binding is key to stopping cellular infiltration of the inflamed site and multiple mechanisms are simultaneously at play to encourage this process. In addition to the molecular regulators described above, chemokines are also simultaneously stimulating diapedesis through the interendothelial spaces along a concentration gradient toward the site of injury or infection where chemokines are actively being produced. Exogenous chemoattractants include bacterial products such as peptides with *N*-formylmethionine as the terminal amino acid and some bacteria-specific lipids while endogenous chemoattractants include a variety of chemokines (such as IL-8), proteins of the complement system (particularly C5a), and arachidonic acid (AA) metabolites (namely LTB_4_). Again, biomaterial approaches have shown an ability to modify these chemokine responses without utilization of drug delivery. In the same Casey article from 2019 (93), in addition to modification of cytokine responses induced my nanoparticle uptake, similarly MCP-1 secretion was shown to be decreased suggesting a global reprogramming of functional responses upon uptake of PLA- and PLGA-based negatively charged particles (Figure 4). In parallel to Moyano et al. (98), modification of chemokines from monocytes can also be achieved with gold-based particles where affected chemokines are dependent on choice of surface coating with lipid-based substrates (99) to decrease chemokine release of IL-8, CCL5/RANTES, and CCL2/MCP-1 vs. decoration of gold particles with peptides containing aromatic and hydrophobic residues to impart a decrease in production of CCL2 and CCL4 (105, 106).

Uniquely, another approach in the literature by Getts et al. (108) bypasses the process of leukocyte migration. Rather than alter the cellular function of inflammatory cells, they showed that PLGA and polystyrene (PS) particles with negatively charged PEMA coating were actively taken up by MARCO^+^ inflammatory monocytes to induce trafficking of these cells away from sites of tissue injury in multiple disease models (including West Nile virus-induced encephalitis, experimental autoimmune encephalomyelitis (EAE), and cardiac infarction). In each of these disease processes, excessive inflammation is implicated as a major source of disease pathogenesis. Whereby uptake of these particles targeted the offending inflammatory monocytes to be actively removed from the circulation and sequestered in the spleen for degradation. As a result of this redirection, this strategy aided in sparing of the end-organs in these disease models most at risk for damage and failure. Another study demonstrated that the composition of nanoparticles, PLGA (high or low molecular weight) vs. PLA, affected their interactions with neutrophils and monocytes *in vitro* and *in vivo*. Using the EAE mouse model, it was demonstrated that high molecular weight PLGA particles significantly improved disease scores compared to controls (107).

**Figure 5:**
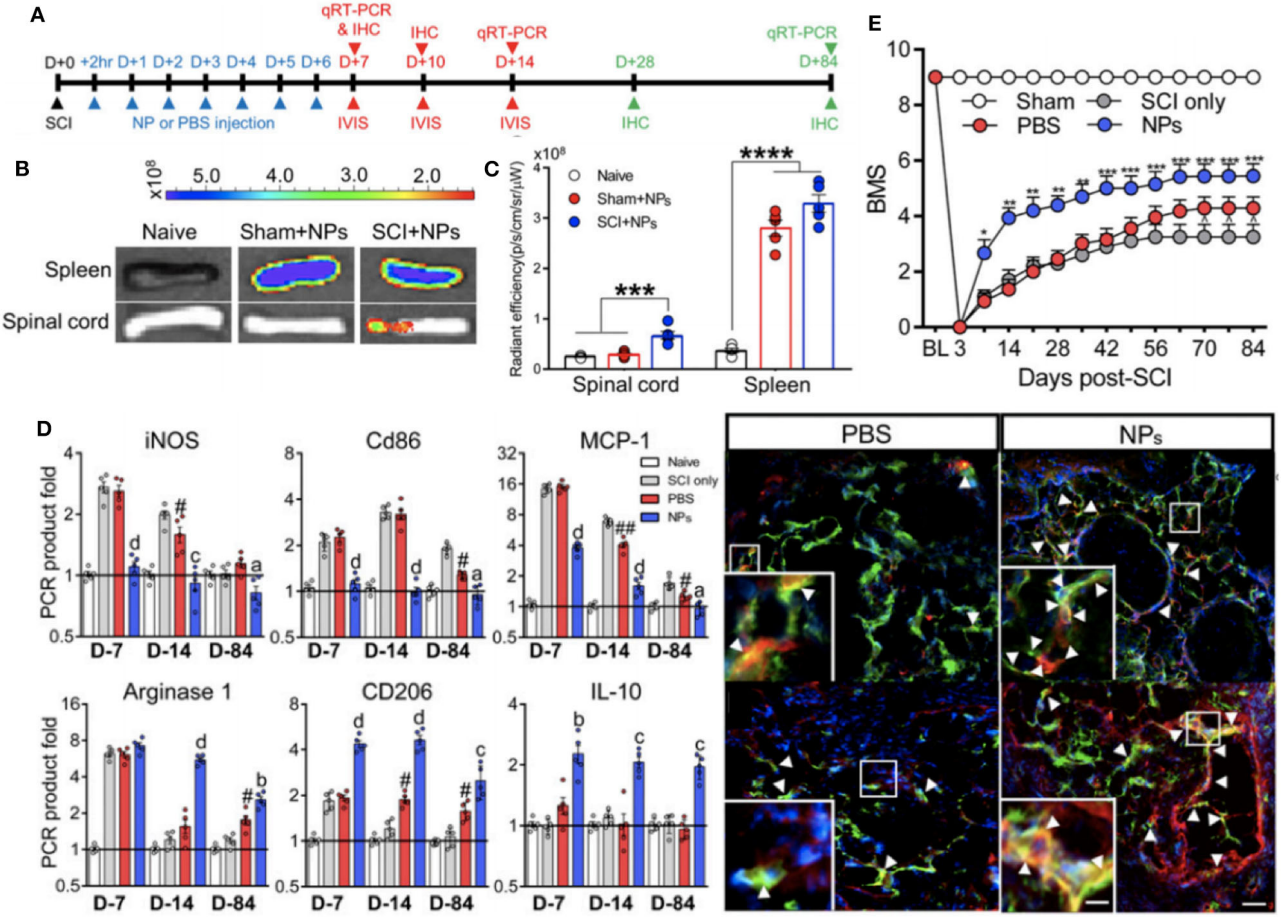
Non-invasive strategy to alter immune cell responses to enhance spinal cord injury (SCI) recovery with *in vivo* biodistribution and analysis of nanoparticles. Experimental timeline for the study **(A)**. *In vivo* images from spinal cord and spleen at 1 day post-injection **(B)**. Fluorescence quantification of imaging in **(B)**, where ***P < 0.001 and ****P < 0.0001 **(C)**. Immunomodulation of macrophages as assessed with RT-qPCR data for pro-inflammatory and anti-inflammatory genes at multiple timepoints post-SCI and immunodetection of M2 macrophages (yellow color) within bridge following SCI (^a^*P* < 0.05, ^b^*P* < 0.01, ^c^*P* < 0.001, and ^d^*P* < 0.0001 compared to the PBS group, and ^#^*P* < 0.05 and ^*##*^*P* < 0.01 relative to the SCI only group) **(D)**. Functional recovery of locomotor activity from SCI, where *P < 0.05, **P < 0.01, and ***P < 0.001 compared the the PBS group, and ^∧^*P* < 0.05 relative to the SCI only group **(E)**. Adapted from (95). Copyright (2019) National Academy of Sciences.

The same nanoparticle approach was taken by Park et al. (95) to abrogate paralysis-induced secondary to traumatic spinal cord injury (SCI). Using the same 500 nm diameter PLGA particles that had been shown to trigger sequestration of inflammatory macrophages and neutrophils away from the injury site (108), a non-invasive strategy was devised to alter the functional capacity of the immune cells at the SCI site and drive a predominantly regenerative phenotype at the SCI (Figure 5). Indeed, as seen with the preceding work, the nanoparticle-containing cells were predominantly sequestered and targeted for destruction at the spleen, but, in combination with spinal cord injury a protective population of M2-like macrophages expressing CD206 selectively homed at the site of injury in a way absent for sham injured mice. With this wound repair phenotype predominating at the SCI site, nanoparticle-mediated promotion of axonal regrowth and remyelination was shown, further emphasizing a therapeutic value to the presence of the materials themselves to engineer the dominant immune response at the site of injury.

### Removing the Offending Microbes and Damaged Tissue: Developing Ways to Limit Collateral Damage

Once neutrophils and monocytes arrive at the injured tissue, recognition of microbes, or dead cells by TLRs and other PRRs drives leukocyte activation with phagocytosis and intracellular killing resulting in clearing of microbes and dead cells (114). As stated in the previous section, however, many of the mechanisms by which these cells clear microbes and dead cells are non-specific and can cause harm to healthy surrounding tissue. Because of this, strategies that can limit this collateral damage in combination with the methods described above can help to alleviate the most destructive organ damage seen with severe inflammation and sepsis.

Phagocytosis requires recognition and attachment by the leukocyte of the agent to be ingested, engulfment of the agent with a phagocytic vacuole, and killing or degradation of the extracellular products taken up by the cell. Recognition is often performed by mannose receptors, scavenger receptors, and a variety of opsonin receptors that can bind and ingest microbes. Of these the macrophage mannose receptor (MMR or CD206) from the lectin family binds terminal mannose and fucose residues of glycoproteins and glycolipids uniquely found on microbial cell walls to drive their phagocytosis (115). This parallels PRR-specific recognition of microbial PAMPs amid ignorance of molecular characteristics of mammalian cells. In a more generalized manner, scavenger receptors constantly sample the environment and can bind and mediate endocytosis of a variety of microbes in addition to oxidized or acetylated low-density lipoprotein (LDL) that fail to interact with the primary LDL receptor. Macrophage integrins, like Mac-1 introduced above (CD11b/CD18) can also bind microbes for phagocytosis. Coating of microbes by opsonins (particularly IgG antibodies, C3b from the complement system, and mannose-binding lectin) greatly increase the efficiency phagocytosis due to high-affinity receptors for opsonins on the cell surface of neutrophils and monocytes (116).

Alteration in CD206 is essential to the phagocytic capability of macrophages and is easily altered by nanoparticle formulations. As described above, Park et al. (95) showed increased levels of CD206, as well as other markers of M2-like macrophages such as IL-10 and arginase-1 at the site of spinal cord injury. In contrast, using peripheral blood monocytes from human volunteers and cynomolgus monkeys, Fruchon et al. (96, 97) show that another nanoparticle formulation using poly(phosphorHydrazone) functionalized with acid azabisphosphorous increased expression of MRC1 complemented with increased cell surface expression of the protein product, CD206.

Once microbes and necrotic debris have been engulfed, final killing and clearance by neutrophils and macrophages requires highly regulated microbicidal activity within phagocytic compartments driven by generation of reactive oxygen and nitrogen species (ROS and nitric oxide, NO, respectively) and lysosomal enzymes (117, 118). ROS production is dependent upon the rapid assembly and activation of NADPH oxidase on the phagosomal membrane. In neutrophils, evolution of superoxide (O_2_·) hydrogen peroxide (H_2_O_2_) is acted upon by myeloperoxidase (MPO) under the influence of halides like Cl^−^ to convert H_2_O_2_ to hypochlorite (${\text{OCl}}_{2}^{-}$). These reactions in combination drive halogenation of microbial components or oxidation of microbial proteins and lipids. In addition to this efficient H_2_O_2_-MPO-halide system, H_2_O_2_ can also be converted to hydroxyl radicals (^−^OH·) to also drive modification of cellular lipids, proteins, and nucleic acids, thus destroying microbes. Similarly, NO is produced from arginine by inducible nitric oxide synthase (iNOS) in macrophages and neutrophils following activation by cytokines (e.g., IFNɤ) or microbial products. NO can then react with O_2_ to form the highly reactive free radical peroxynitrite (ONOO^−^) to damage the lipids, proteins, and nucleic acids of microbes in a manner similar to ROS. Additional intracellular microbicidal activity is driven by lysosomal enzymes contained in lysosomal granules that contribute to microbial killing and vast amounts of tissue damage.

Although multiple groups have shown an ability to decrease ROS production *in vitro* (98, 100), Soh et al. (119) introduce an interesting twist in monocytes by using ceria-zirconia nanoparticles to actively scavenge ROS given the faster conversion of ceria-zirconia nanoparticles to convert between the Ce^3+^ and Ce^4+^ oxidation states of ceria nanoparticles alone. In LPS-induced endotoxemia rat models and CLP-induced bacteremia mouse models, this increase in ROS and NO scavenging had a net effect of increasing animal survival. At a tissue level, this increased survival is correlated to sparing of the liver and lungs from LPS-associated ROS and NO immune damage with intravenous LPS administration and the gastrointestinal tract of damage associated with similar bactericidal immune mechanisms in widespread polymicrobial bacteremia.

Additional mechanisms at play in microbial killing include neutrophil extracellular traps (NETs) that are composed of extracellular fibrillar networks with a high concentration of antimicrobial substances at the site of infection. These have the ability to trap microbes within the fibrils in response to bacteria and fungi and inflammatory mediators such as cytokines and chemokines, complement proteins, and ROS. NETs are viscous in nature due to neutrophil nuclei loss during NET formation leading to extracellular chromatin binding and concentrating granule proteins and these NETs have been shown in the literature to be particularly destructive during sepsis as they are broken down (120). Recent murine work has shown that antibody-mediated stabilization of NETs prevents release of their captured bacteria and additional toxic NET contents has shown to be protective during sepsis (121), suggesting further opportunities to design biomaterials to aid in minimizing in a controlled fashion the deleterious effects of this necessary microbicidal mechanism.

### Resolution of Inflammation

Given the powerful host defense mechanisms at play during inflammation, resolution of the response needs to be tightly controlled to prevent deleterious consequences. Although complete resolution of inflammation is ideal, other consequences of inflammation include connective tissue replacement for healed tissues (scarring or fibrosis) and chronic inflammation.

Among endogenous modulators of inflammation, many are closely related to those driving the inflammatory response. Another AA metabolite class, lipoxins, serve to aid in resolution of inflammation by preventing leukocyte recruitment. LXA_4_ and LXB_4_ serve to prevent neutrophil chemotaxis and adhesion during the presence of both neutrophils and platelets at the site of inflammation. Among cytokines, transforming growth factor-beta (TGF-β) and IL-10 are generally regarded as having anti-inflammatory activity. With some nanoparticle strategies, direct induction of IL-10 production (55, 94, 96, 97, 101) has been possible with a variety of biomaterial composition approaches (Table 4). Additionally, the complement system contains a number of regulatory components with even more soluble protein mediators of resolving inflammation include resolvins, protectins, and maresins (129).

**Table 4:** Empirical relationships determined between biomaterial physicochemical properties and immune cell activity.

**Physicochemical properties**				**Immunological variables**			**References**
**Composition**	**Size**	**Zeta potential/charge**	**Surface coating/functionalization**	**Cell type**	**Animal models**	**Biological effect**	
**Polymers**							
Poly(*N*-isopropylacrylamide)	80–300 nm	Neutral charge, hydrophobic		Erythrocytes	Human	Sequestration and neutralization of lipid-based toxins with erythrocyte sparing	(74, 75)
Poly(lactic acid)	350–500 nm	−50 to −40 mV	Poly(ethylene-*alt*-maleic acid)	Bone marrow-derived macrophages	Mice (C57BL/6)	Decreased secretion of IL-6, TNF-α, and MCP-1; decreased expression of MHC II, MARCO, CD80, CD86	(93)
Poly(lactic acid)	430–470 nm	−47 to −31 mV	Poly(ethylene-*alt*-maleic acid)	Inflammatory monocytes, neutrophils	Mice (SJL/J)	Negligible sequestration of inflammatory monocytes and neutrophils away from sites of inflammation	(107)
Poly(lactic acid)	350–500 nm	−25 mV	Poly(vinyl alcohol)	Bone marrow-derived macrophages	Mice (C57BL/6)	Partial decreased secretion of IL-6, TNF-α, and MCP-1	(93)
Poly(lactic-*co*-glycolic acid)	100–104 nm	−7 to −5 mV		Neutrophils	Human	Cationic surfactant leads to dramatic neutrophil death and LDH release	(122)
Poly(lactic-*co*-glycolic acid)	100–105 nm	−30 to −23 mV	Macrophage cell membrane	J774 macrophages, human umbilical vein endothelial cells	Mice (C57BL/6, BALB/c)	Sequestration of LPS; sequestration of IL-6, TNF-α, IFNɤ; decreased iNO production; decreased E-selectin expression; decreased serum IL-6, TNF-α, IFNɤ; survival benefit in *E. coli* bacteremia	(54)
Poly(lactic-*co*-glycolic acid)	140–165 nm	0 to 0.5 mV	di(α2 → 8)*N*-acetylneuraminic acid	Peritoneal macrophages	Mice (C57BL/6)	Decreased secretion of TNF-α, IL-6; increased Siglec-E expression; increased serum IL-10; decreased serum IL-6; increased survival benefit in LPS-induced endotoxemia	(94)
Poly(lactic-*co*-glycolic acid)	214–226 nm	35 to 43 mV	Soyaethyl morpholinium ethosulfate	Neutrophils	Human	Cationic surfactant leads to dramatic neutrophil death and LDH and elastase release, moderate increase in superoxide production	(122)
Poly(lactic-*co*-glycolic acid)	240–252 nm	24 to 34 mV	Cetyltrimethylammonium bromide	Neutrophils	Human	Cationic surfactant leads to dramatic neutrophil death and LDH and elastase release, dramatic increase in superoxide production	(122)
Poly(lactic-*co*-glycolic acid)	350–500 nm	−50 to −40 mV	Poly(ethylene-*alt*-maleic acid)	Bone marrow-derived macrophages	Mice (C57BL/6)	Decreased secretion of IL-6, TNF-α, and MCP-1; decreased expression of MHC II, MARCO, CD80, CD86; survival benefit in LPS-induced endotoxemia	(93)
Poly(lactic-*co*-glycolic acid)	350–500 nm	−25 mV	Poly(vinyl alcohol)	Bone marrow-derived macrophages	Mice (C57BL/6)	Partial decreased secretion of IL-6, TNF-α, and MCP-1	(93)
Poly(lactic-*co*-glycolic acid)—low molecular weight	330–510 nm	−56 to −40 mV	Poly(ethylene-*alt*-maleic acid)	Inflammatory monocytes, neutrophils	Mice (SJL/J)	Negligible sequestration of inflammatory monocytes and neutrophils away from sites of inflammation	(107)
Poly(lactic-*co*-glycolic acid)—high molecular weight	510–590 nm	−43 to −36 mV	Poly(ethylene-*alt*-maleic acid)	Inflammatory monocytes, neutrophils	Mice (C57BL/6, SJL/J, BALB/c)	Sequestration of inflammatory monocytes and neutrophils away from sites of inflammation	(107)
Poly(lactic-*co*-glycolic acid)	500 nm	Negatively charged	Poly(ethylene-*alt*-maleic acid)	MARCO^+^ macrophages	Mice (C57BL/6, SJL/J, BALB/c)	Sequestration of inflammatory monocytes away from sites of inflammation; functional reprogramming of macrophages from M1 to M2 at site of spinal cord injury	(95, 108)
Polystyrene	100–117 nm		Denatured albumin	Neutrophils	Mice	Albumin nanoparticles taken up by activated neutrophils through endocytosis mediated with FcɤRIII to reduce neutrophil adhesion and migration across the endothelium to limit vascular inflammation modulated via adherent neutrophils; protective against lung injury with Syk inhibitor to prevent inside-out signaling	(102, 103)
Polystyrene	500 nm	Negatively charged	Poly[ethylene-*alt*-maleic acid)	MARCO^+^ macrophages	Mice (C57BL/6, SJL/J, BALB/c)	Sequestration of inflammatory monocytes away from sites of inflammation	(108)
**LIPIDS**							
Liposomes−3.2% soybean phosphatidylcholine and 0.8% cholesterol	51–60 nm	37 to 55 mV	Cetyltrimethylammonium bromide	Neutrophils	Human	Dramatic neutrophil death, LDH release, high superoxide production, Ca^2+^ mobilization, promptly induces NET formation	(123)
Liposomes−3.2% soybean phosphatidylcholine and 0.8% cholesterol	73–81 nm	19 to 36 mV	Soyaethyl morpholinium ethosulfate	Neutrophils	Human	Increased neutrophil death at increasing concentrations, LDH release at high concentrations of surfactant	(123)
Liposomes−3.2% soybean phosphatidylcholine and 0.8% cholesterol	88–92 nm	−49 to 39 mV		Neutrophils	Human	Inert for neutrophils *in vitro*	(123)
Solid lipid nanoparticles (SLNs)−12% cetyl palmitate and 1% soybean phosphatidylcholine	192 nm	−41 mV		Neutrophils	Human	Inert for neutrophils *in vitro*	(124)
Solid lipid nanoparticles (SLNs)−12% cetyl palmitate and 1% soybean phosphatidylcholine	195 nm	44 mV	Cetyltrimethylammonium bromide	Neutrophils	Human	Dramatic neutrophil death, LDH release, superoxide production, elastase release, Ca^2+^ mobilization, p38 and JNK activation, and NET development	(124)
Nanostructured lipid carriers (NLCs)—composed of both solid and liquid lipids with a soft core matrix of 6% w/w soybean oil, 65% cetyl palmitate, 1% soybean phosphatidylcholine (SPC)	162–177 nm	51 to 53 mV	Cetyltrimethylammonium bromide	Neutrophils	Human	Dramatic neutrophil death and LDH and elastase release, moderate increase in superoxide production	(122)
Nanostructured lipid carriers (NLCs)—composed of both solid and liquid lipids with a soft core matrix of 6% w/w soybean oil, 65% cetyl palmitate, 1% soybean phosphatidylcholine (SPC)	248–261 nm	−44 to −41 mV		Neutrophils	Human	Inert for neutrophils *in vitro*	(122)
Nanostructured lipid carriers (NLCs)—composed of both solid and liquid lipids with a soft core matrix of 6% w/w soybean oil, 65% cetyl palmitate, 1% soybean phosphatidylcholine (SPC)	257–261 nm	51 to 52 mV	Soyaethyl morpholinium ethosulfate	Neutrophils	Human	Cationic surfactant leads to dramatic neutrophil death and LDH release	(122)
**DENDRIMER**							
Poly(phosphorHydrazone)			Acid azabisphosphorous	Monocytes	Human volunteers, cynomolgus monkeys	Increased expression of *MRC1, IL1RN, IL10, CCL18, CD23, CCL5*; increased expression of cell surface CD206, decreased cell surface expression of CD64, CD13, HLA-DR, HLA-A/B/C, CD86	(96, 97)
**METALS/METAL OXIDES**							
Gold	2 nm (core)	Neutral charge	Tetraethylene glycol with end hydroxyl group	Monocytes	Mice	Decreased ROS production *in vitro*, decreased TNF-α production *in vitro*; decreased serum TNF-α following LPS-induced endotoxemia	(98)
Gold	2 nm (core)	Neutral charge, hydrophobic	Tetraethylene glycol with hydrophobic end group	Monocytes	Mice	Decreased ROS production *in vitro*, decreased TNF-α production *in vitro*; increased serum TNF-α following LPS-induced endotoxemia	(98)
Gold	2 nm (core)	Neutral charge, hydrophilic	Tetraethylene glycol with hydrophilic end group	Monocytes	Mice	No change over LPS treatment alone *in vitro* or *in vivo*	(98)
Gold	5 nm		Inner lipid: 1,2-dipalmitoyl-*sn*-glycero-3-phosphoethanolamine-N-[3-(2-pyridyldithio)propionate] (PDP PE 16:0) or 1,2-dioleoyl-*sn*-glycero-3-phosphoethanolamine-N-[3-(2-pyridyldithio)propionate (PDP PE 18:1) Outer lipid: 1,2-dipalmitoyl-*sn*-glycero-3-phsphocholine (DPPC), spingomyelin, cardiolipin, 1,2-dilinoleoyl-*sn*-glycero-3-phospho-(1'-rac-glycerol) (18:2 PG), 1,2-dimyristoyl-*sn*-glycer-3-phosphoethanolamine-N-(lissamine rhodamine B sulfonyl) (14:0 Liss Rhod PE)	Monocytes	Human	Decreased NF-κB activation; decreased expression of *Il1b*; decreased secretion of IL-1β, TNF-α, IL-6, IL-8, CCL5/RANTES, CCL2/MCP-1, GM-CSF	(99)
Gold	13–20 nm	Negative charge	Peptides with hydrophobic and aromatic residues	Monocytes		Decreased NF-κB and IRF3 activation following TLR agonist treatment, decreased CCL2 and CCL4 production; decreased lung damage and survival benefit in LPS-induced ALI; larger particles are more protective	(105, 106)
Silver	4 nm	−25 to −8 mV	Poly(vinyl alcohol)	Neutrophils	Human	Induces apoptosis and increases ROS generation at high concentrations (50 μM)	(125)
Silver	10 nm		Poly(vinyl pyrrolidone)	Neutrophils	Human	Increased cell death at greater concentrations (range of 25–100 μg/mL) with corresponding increases in neutrophil oxidative burst	(126)
Silver	15 nm	−9 to −7 mV		Neutrophils	Human	Atypical cell death at low concentrations (≤25 μg/mL) with no CD16 shedding, caspase-1 and caspase-4 dependent IL-1β activation, and caspase-1 and caspase-4 independent NET formation; necrosis at high concentrations (>50 μg/mL)	(127)
Silver	20 nm	−11 to −8 mV		Neutrophils	Human	High concentrations (100 μg/mL) induce apoptosis of neutrophils and inhibition of *de novo* protein synthesis	(128)
Silver	50 nm		Poly(vinyl pyrrolidone)	Neutrophils	Human	Limited cell death at greater concentrations (range of 25–100 μg/mL)	(126)
Ceria-zirconia (Ce_0.7_Zr_0.3_O_2_)	2–4 nm			Monocytes	Rats, mice	Antioxidant activity (SOD, catalase, CAT, mimetic and hydroxyl radical antioxidant capacity, HORAC) decreased; decreased LDH; decreased CD68^+^ monocytes at site of injury; survival benefit in LPS-induced endotoxemia and CLP	(119)
**CARBON-BASED**							
Carbon nanotube	0.4–4.2 nm (length), 12–34 nm (diameter)		Carboxymethyl cellulose	Macrophages		Sequestration of complement proteins triggering opsonization	(57)
Carbon nanotube	0.4–4.2 nm (length), 12–34 nm (diameter)		RNA	Macrophages		Sequestration of complement proteins triggering opsonization	(57)
Hydroxylated fullerene (C_60_[OH]_44_)				Peritoneal macrophages	Mice (C57BL/6)	Decreased ROS production; decreased expression of *Il6, Il1b, Tnf*; decreased preterm birth	(100)
Nanodiamond	5 nm	Negatively charged	Octadecylamine	Macrophages	Human	Decreased TNF-α, IL-1β secretion and increased IL-10 secretion	(101)
**BIOMIMETIC**							
Cell-derived nanoparticle (CDNPs)—composed of annexins, actin, histones, heat shock proteins, myosin, peroxiredoxines and vimentin and small traces of nucleic acids, with annexin A5 (AnxA5) being one of the most abundant components; [protein] = 150 μL/mL, [DNA] = 2 μg/mL, [RNA] = 4 μg/mL	50–200 nm			Source of CDNPs: MC3T3-E1 fibroblast cells, peritoneal lavage Takes up CDNPs: neutrophils, inflammatory monocytes, macrophages	Mice	Decreased peritoneal IL-6 and IL-10 following CLP; decreased bacterial growth *in vitro*; increased expression of CD11b and MHCII on the cell surface of neutrophils, inflammatory monocytes, and macrophages	(55)
Cell membrane-formed nanovesicles (cell disruption by nitrogen cavitation, centrifugation, and extrusion—contains integrin β2	200 nm	−20 to −12 mV		Source of nanovesicles: HL 60 myeloid cells		Binds ICAM-1 on HUVEC to deliver NF-κB inhibitors and stop diapedesis	(104)

Because of the destructive nature of lysosomal enzymes, antiproteases are also present in the serum and tissue fluids to limit inflammation-associated lysosomal damage. Of these, α_1_-antitrypsin is a major inhibitor of neutrophil elastase and α_2_-macroglobulin is another found in serum and various secretions. Additionally, neutrophils themselves have very short lives and turnover of inflammatory cells and the produced mediators of inflammation following removal of the provoked injury are key to resolution. Of note, however, is that in cases of sepsis neutrophil apoptosis is delayed but their function is impaired. Under normal conditions circulating neutrophil have a short half-life (7–12 h *in vivo*) but this is increased downstream of LPS- and C5a-mediated neutrophil activation. This is attributed to a combination of pro-survival cell signaling, including decreased activation of caspase-8 (130) leading to an accumulation of nuclear factor myeloid nuclear differentiation antigen (MNDA) in parallel with accumulation of Mcl-1 (131), increased anti-apoptotic Bcl-xL (132), decreased pro-apoptotic Bim (133), and increased phosphorylation of Bad downstream of Akt activation (134–136). The net result of these combined molecular mediators is decreased neutrophil apoptosis. This long-lived neutrophil population in sepsis is also characteristic for its impaired transmigration to the site of inflammation. Rather than limiting the damaging effects of neutrophils solely to the site of tissue microbes and injury, neutrophils in sepsis are marked by aberrant neutrophil localization into remote organs where they can inflict damage and further augment the damage of inflammation (137–139).

Given the dramatic destruction inflicted by dysregulated trafficking of long-lived neutrophils during sepsis, it is of benefit to generate therapeutic strategies that can eliminate neutrophils while minimizing the collateral damage inflicted by these cells (behaving as they are expected to do) in aberrant tissue sites. As such, strategies in the literature that were originally intended as studies of the toxicity of nanoparticles provide hints of ways to normalize neutrophil behavior and limit organ dysfunction. With this in mind, Table 4 reiterates the studies discussed above and summarized in Tables 1–3 with an emphasis on cataloging features in biomaterial design. This allows for emphasizing the relationship between the physicochemical characteristics of the chosen materials and the resultant biological effects from the perspective of immune responses at the cellular and, when available, animal model level. As an example of how fine-tuning of physicochemical properties can be harnessed for desired biological effects, the Girard Lab provides an elegant series of studies that stresses this point. This group has shown (127, 128) with human neutrophils that silver nanoparticles in the range of 15–20 nm induced apoptosis and atypical cell death of neutrophils with the ability to inhibit *de novo* protein synthesis. In related studies, silver nanoparticles were further coated with either PVA (125) or poly(vinyl pyrrolidone) (PVP) (126) to show a size-dependence to cell death induction. Indeed, smaller nanoparticles (4–10 nm) showed the most dramatic cell death in a manner dependent on neutrophil oxidative burst, while even small variations in nanoparticle size (50 nm) abrogated the neutrophil cell death. As this series of studies tell us, each element of material design, from the core material to size to even the choice of surfactant, can impart a dramatic change in the functional responses of innate immune cells further highlighting the importance of cataloging physicochemical characteristics to enable rational design strategies for immunomodulation.

## Polypharmacological Strategies for Severe Inflammation and Sepsis

The dysregulation that develops due to sepsis affects cellular phenotypes and gene expression profiles in both transient and long-term manners. In humans, LPS administration resulted in 3,714 genes being differentially expressed in blood leukocytes as early as 2 h post exposure with a near complete resolution of clinical perturbations within 24 h post challenge (140). Similar genomic studies in mice corroborate the vast genetic alterations and have identified over 1,900 differentially expressed genes following LPS challenge (141). Sepsis survivors generally suffer from additional morbidities including higher risk of readmission, cardiovascular disease, cognitive impairment, and death for years following sepsis. Epigenetic mechanisms such as DNA methylation, histone modifications, and non-coding RNAs are also perturbed in sepsis and are associated with increased mortality due to their contributions to long-term immunosuppression (142). Given that thousands of genes are differentially expressed during sepsis, the number of tractable therapeutic options that aim to augment or abrogate single molecular targets is out of the scope of practical and experimental possibilities.

Multiple target-based approaches should be considered to improve patient outcomes in sepsis. A single timepoint nor single cytokine/receptor intervention is unlikely to be successful on a broad range of patients with diverse conditions that have led to the state of sepsis (143). The complexity of disease states offers a range of potential molecular targets, as well as numerous other factors including the time of treatment administration and the combination of drugs. Providing further evidence for multi-target approaches, Cockrell and An developed computational algorithms and predicted the necessity for a multi-target therapy for the treatment of sepsis (144). The specificity to which small molecules and biologics modulate immune responses at a single-target level or through non-specific mechanisms limits their utility in treating the underlying dysfunction encoded in immune cells during and following sepsis. Due to the lack of conceivable small molecules or biologics, nanoparticles are uniquely positioned to achieve this goal due to their highly controllable physicochemical properties, targetability, and immune-modulating properties (7).

A polypharmacological strategy has the potential to address the redundant molecular, cellular, and tissue functions during inflammation but anti-inflammatory and anti-coagulants are neither innocuous nor without potential adverse effects in combination or alone. Of particular note is that morbidity and mortality associated with sepsis and septic shock tends to be most severe within the geriatric and pediatric population, two groups where polypharmacy can be especially deleterious in combination with existing comorbidities or developmental concerns (145–149). Given these concerns with a multi-drug approach, other strategies that can work with multimodal mechanisms of action and minimize adverse effects are ideal and ongoing research with biomaterials serves as an exciting area to deliver on some of these strategies. As such, biomaterials and what is known about the cellular and tissue effects of their physicochemical properties will serve as the focus of the remainder of this review.

There are advantages and disadvantages to each strategy in the management or cure of disease. However, particularly noteworthy in polypharmacology is the reduction in treatment complexity, reduced side effects, and reduced or altogether eliminated drug–drug interactions, in addition to improved patient compliance. Also, given that a single agent can simultaneously affect multiple targets in the same tissue (by default, both pharmacophores must co-localize), partial modulation of targets that are synergistically linked suggests that reduced doses may be sufficient to elicit full therapeutic efficacy, widening the therapeutic windows.

## Conclusion and Future Prospects for Biomaterial-Driven Immune Modulation

Developing strategies to control severe inflammation and sepsis remains a healthcare priority. Given the toll sepsis and septic shock plays in increasing healthcare costs and the continuing staggering rates of mortality and long-term morbidity for those affected, it is essential that strategies to improve patient outcomes are informed by the pathophysiology of dysregulated inflammation. As laid out in the review, although sepsis can be triggered by one of numerous types of bacteria breeching initial defenses at a variety of tissue sites, the course of inflammation itself, although complex, has stereotypic physiological processes that provide opportunities for intervention (Figure 1). From the survey of studies included in this review, diverse strategies have been implemented that attempt to address each stage: (1) limiting initial activation of innate immune cells (Figure 2 and Table 1), (2) regulating pro-inflammatory mediators (Figures 3, 4 and Table 2), (3) inhibiting further leukocyte recruitment (Figures 1, 4, 5 and Table 3), (4) removing the initiating microbe and signals for inflammation, and (5) regulating mediators of resolution (Figures 3, 4 and Table 2). Among these works, the strategies with the most promise are those that attempt to affect multiple stages of this process. Indeed, the complex and parallel physiologic responses that have been thus far accounted for during sepsis show that effective management of sepsis requires a multi-targeted approach.

As we have put forth, biomaterials and the generation of nanotechnology-based approaches has the potential to allow for finely tuned engineering of immune responses based on experimentally determined rational design principles. Through elucidation of the principles at play in development of these biomaterials and nanoparticle platforms, the potential exists to generate multi-targeted therapeutics that meet our specific needs based on physicochemical properties deemed significant (e.g., composition, size, charge, and others) as summarized in Table 5. With the maturation of nanotechnology-based immune engineering, several outstanding questions remain to be addressed by all stakeholders in the field including development of biologically relevant animal models, standardization of GMP manufacturing procedures, standardization of formulations with potential implications for pharmacokinetics and pharmacodynamics, and further guidance from regulatory agencies in regard to the nanocarriers themselves. It is our hope that in the upcoming years, these design principles are further developed and adopted in the field as these questions for scalability of nanotechnology are addressed. Future biomaterial designs will be informed by the immunology it intends to assist and, vice versa, the immunology continues to provide new avenues of exploration for the application of biomaterials to improve human health. This interface promises to expand the development of nano-based therapeutics as well as to further the basic understanding of nano-bio interactions and their implications for therapeutic strategies.

**Table 5:** Nanoparticle physicochemical properties and desired immune responses to consider when designing biomaterials to fine tune inflammatory responses.

**Tunable nanoparticle physicochemical properties**	
**Chemical composition**	**Nanoparticle diameter**
• **Nanoparticle core** ° Polymer ° Lipid ° Metal/metal oxide ° Carbon-based ° Biomimetic • **Surfactant** ° Polymer-based ° Carbon-chain length ° Biomimetic source • **Functionalization** ° Core polymer conjugations ° Additional small molecules or biologics	• <200 nm • 200–800 nm • >800 nm **Surface properties** • **Zeta potential/charge** ° <-25 mV/negative ° −25 to 10 mV/neutral ° >10 mV/positive • **Surface chemistry** ° Hydrophilic ° Hydrophobic ° Zwitterionic
**Desired nanoparticle-mediated immunological outcomes**	
**Recognition of microbes/PAMPs/DAMPs**	
• Prevention of recognition by immune cells • Sequestration of insults away from immune site	
**Regulators of inflammation**	
• Alteration of transcription factor activity • Prevention of inflammatory gene activation • Prevention/delay of cytokine and chemokine release • Tuning of inflammatory mediators at immune site	
**Leukocyte recruitment**	
• Activation state of vascular endothelium • Sequestration of immune cell subsets away from immune site • Recruitment of immune cell subsets to immune site	
**Managing immune-mediated tissue damage**	
• Scavenging of ROS and RNS • Modification of NET stability	
**Resolution of inflammation**	
• Alteration of programmed apoptotic pathways • Alteration of phagocytic capacity • Increased clearance rate of pro-inflammatory regulators	

## Author Contributions

JL and RP conceptualized the manuscript, wrote the text, drew and adapted figures and tables, and approved the manuscript. HK and MM assisted with gathering, reviewing references, and approved the manuscript. All authors contributed to the article and approved the submitted version.

## Conflict of Interest

The authors declare that the research was conducted in the absence of any commercial or financial relationships that could be construed as a potential conflict of interest.
